# Adipose Tissue Immune Response: Novel Triggers and Consequences for Chronic Inflammatory Conditions

**DOI:** 10.1007/s10753-014-9914-1

**Published:** 2014-05-14

**Authors:** Giorgio Ghigliotti, Chiara Barisione, Silvano Garibaldi, Patrizia Fabbi, Claudio Brunelli, Paolo Spallarossa, Paola Altieri, Gianmarco Rosa, Giovanni Spinella, Domenico Palombo, Razvan Arsenescu, Violeta Arsenescu

**Affiliations:** 1 Division of Cardiology, IRCCS University Hospital San Martino, Research Centre of Cardiovascular Biology, University of Genoa, Genoa, Italy; 2 Vascular and Endovascular Surgery Unit, University of Genoa, Genoa, Italy; 3 IBD Center, Division of Gastroenterology, Hepatology and Nutrition, The Ohio State University, Columbus, OH USA; 4 Mucosal Immunology IBD Laboratory, Division of Gastroenterology, Hepatology and Nutrition, The Ohio State University, 400W 12 Ave., Wiseman Hall, Room 1024, Columbus, OH 43210 USA

**Keywords:** adipose tissue, inflammation, macrophage, NK, NKT, eosinophil, neutrophil, adiponectin, angiotensin, aryl hydrocarbon receptor, abdominal aortic aneurysm, mast cell inflammatory bowel disease, cardiorenal syndrome, chronic inflammatory diseases, uric acid

## Abstract

Adipose tissue inflammation mediates the association between excessive body fat accumulation and several chronic inflammatory diseases. A high prevalence of obesity-associated adipose tissue inflammation was observed not only in patients with cardiovascular conditions but also in patients with inflammatory bowel diseases, abdominal aortic aneurysm, or cardiorenal syndrome. In addition to excessive caloric intake, other triggers promote visceral adipose tissue inflammation followed by chronic, low-grade systemic inflammation. The infiltration and accumulation of immune cells in the inflamed and hypertrophied adipose tissue promote the production of inflammatory cytokines, contributing to target organ damages. This comorbidity seems to delimit subgroups of individuals with systemic adipose tissue inflammation and more severe chronic inflammatory diseases that are refractory to conventional treatment. This review highlights the association between adipose tissue immune response and the pathophysiology of visceral adiposity-related chronic inflammatory diseases, while suggesting several new therapeutic strategies.

## INTRODUCTION

Excessive body fat is a chronic inflammatory disorder that affects people of all ages and ethnicities. Worldwide, illnesses related to excess adipose tissue have emerged as the leading causes of cardiovascular mortality [1]. Obese individuals have higher prevalence of hypertension, diastolic dysfunction, left ventricular hypertrophy, increased arterial stiffness, and arterial calcification compared to normal weight individuals [2, 3]. In overweight and obese children, hemodynamic alterations and abnormal metabolic parameters can be present even at very young age and are silently working their way toward chronic inflammatory diseases [4–8]. Moreover, the increased periorgan fat mass can have direct functional and mechanical roles contributing to the subclinical organ damage by exerting local toxic effects [9, 10]. In the general population, excessive adipose tissue is associated with high incidence of nonalcoholic fatty liver disease (NAFLD) [11], chronic kidney disease [12], and end-stage renal failure [13]. Adipose tissue immune response to various triggers is different based on anatomical location. Visceral adipose tissue mass is a major determinant of endothelial dysfunction, liver steatosis, plasma level of adiponectin, atherosclerosis, and metabolic syndrome [14]. Importantly, inflammation appears to be a common denominator for all of these abnormal metabolic conditions [15].

**Fig. 1:**
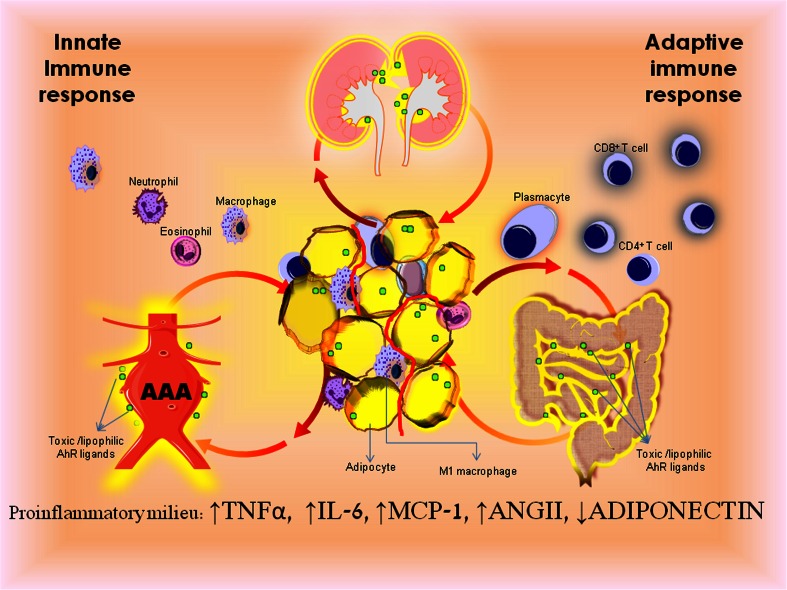
Obesity-induced visceral fat inflammation promotes end-organ chronic inflammatory damage. Obesity-related adipose cell dysfunction triggers migration of innate and adaptive immune effector cells. Activation of immune system and adipose cell dysfunction promotes an inflammatory milieu characteristic to obesity-related pathologic states. Chronic production of TNF-α, IL-6, and MCP-1 and an increased ratio of angiotensin II to adiponectin maintain a vicious pathologic cycle that culminates in organ damage. Accumulation of dioxin-like environmental toxicants (AhR ligands) in adipose tissue amplifies diet-related adipocyte hypertrophy (as seen in abdominal aortic aneurysm, inflammatory bowel diseases or cardiorenal syndrome).

Adipose tissue is an organ enriched in macrophages and capable of generating and sustaining a strong inflammatory response to noxious triggers by involving both the adipocytes and the vascular stroma fraction (25 and 75 % of the adipose tissue cell population, respectively) [16]. Several mechanisms are involved in adipocyte inflammation: (1) adipocyte stress response (hypertrophy, hypoxia, and endoplasmic reticulum stress), (2) altered adipokine secretion, and (3) adoption of a macrophage-like phenotype. These events are interrelated and can sustain each other. Adipocyte hypertrophy has been shown to be causally linked with inflammation and systemic insulin resistance. Increased adipocyte size changes the adipocyte membrane capacity to adapt to adipose tissue expansion, potentially leading to higher vulnerability to inflammation [17]. In obese Zucker rats, adipocyte hypertrophy is followed by a proportional increase in the adipocyte lipid droplet size and a higher concentration of caveolin-1 onto each lipid droplet surface. The caveolin-1-dependent endothelium pathway has been shown to participate in the control of macrophage extravasation from the blood into the adipose tissue [18]. This inflammatory milieu triggers intrinsic inflammatory molecules like tumor necrosis factor alpha (TNF-α), which can either sustain a macrophage-like phenotype in undifferentiated precursor cells [19] or diminish the ability of mature adipocytes to further expand and store lipids, thus sustaining an already “vicious cycle” (Fig. 1). Other proinflammatory chemokines, such as monocyte chemoattractant protein-1 (MCP-1/CCL2) and its receptor (CC chemokine receptor 2 (CCR2)), are highly expressed on hypertrophied adipocytes and can accelerate the migration of the bone marrow-derived monocytes/macrophages and their adipose tissue homing [20]. Autocrine and paracrine regulatory loops involving angiotensin and adiponectin can further modulate the cross talk between adipocytes and adipose tissue macrophages [21, 22]. Senescence, necrosis, and adipocyte death are associated with increased macrophage infiltration in the expanded adipose tissue (Fig. 1).

Differential activation of adipose tissue macrophages modulates the amplitude of adipose tissue inflammation. Depending on the types of stimuli, macrophages respond with either classic proinflammatory (M1) or alternative anti-inflammatory (M2) activation. Under normal physiologic circumstances, the adipose tissue-resident macrophages exhibit an alternatively activated, reparative, or M2 phenotype. Enlarged and dysfunctional adipocytes favor and sustain the activation of classic proinflammatory macrophages or the M1 phenotype [23] that will further arrest the recruitment of healthy, small fat cell progenitors. In time, and due to a limited vascular supply, the hypertrophied mature adipocytes will become fibrotic and drive subclinical inflammation toward chronic irreversibility [24].

## ADIPONECTIN AND ANGIOTENSIN CROSS TALK IN ADIPOSE TISSUE INFLAMMATION

Adipose tissue produces several adipokines with important roles in adipose tissue metabolism, inflammation, as well as systemic effects on other organs [25]. Adiponectin is the main anti-inflammatory mediator produced in adipose tissue [26]. Human adiponectin gene contains a signal sequence, a collagen-like domain, and a globular domain similar to the complement factor C1q. Biological effects of adiponectin depend upon the formation of multimeric complexes. The basic unit is a trimer, which can associate through disulfide bonds to generate hexamers and dodecamers referred to as low, medium, and high molecular weight adiponectin (LMW, MMW, HMW), respectively. Cleavage of the adiponectin molecule by leukocyte esterase can release the globular part, which retains biological activity. It is important to distinguish between these isoforms since they may have opposing effects on inflammation [27, 28]. Both proinflammatory and anti-inflammatory effects have been described for all forms of adiponectin. This is in part explained by the experimental conditions and cell type, although lipopolysaccharide (LPS) contamination is another important factor. Recent studies suggest that HMW adiponectin is the main anti-inflammatory moiety. *In vitro* experiments have shown that globular adiponectin induces nuclear factor kappa B (NF-kB) and proinflammatory cytokines, but prolonged exposure blocks further activation. In contrast, HMW adiponectin can quickly prevent NF-kB activation.

Adiponectin production is regulated at transcriptional and posttranslational levels [29]. During adipogenesis, several transcription factors, including peroxisome proliferator-activated receptor gamma (PPARγ), bind its promoter to upregulate adiponectin messenger RNA (mRNA) expression. Plasma level of adiponectin is negatively correlated with body mass index (BMI) and visceral fat accumulation [30]. Therefore, obese and morbidly obese patients have low and very low adiponectin levels, respectively. Weight loss through caloric restriction, exercise, or bariatric surgery increases adiponectin and/or the ratio of HMW to total adiponectin [31, 32]. *In vivo* and *in vitro* studies suggest that the visceral rather than the subcutaneous fat is the main source of adiponectin. Importantly, the size of adipocytes correlates with the amount of secreted protein. Large, mature, and insulin-insensitive adipocytes secrete very little adiponectin in comparison with small, young, and insulin-sensitive preadipocytes. Isakson et al. isolated fresh, mature adipocytes from obese individuals and showed that they had an increased expression of mitogen-activated protein 4 kinase 4 (MAP4K4), which is known to inhibit peroxisome proliferator-activated receptor gamma (PPARγ) induction and the recruitment of new, small insulin-sensitive preadipocytes [19].

Two main adiponectin receptors have been identified, with homology to G protein-coupled receptors. These receptors have distinct tissue specificities within the body and have different affinities to the various forms of adiponectin (monomers or multimers). Adiponectin binds to the extracellular –COOH terminus of adiponectin receptors (AdipoR1/AdipoR2) and recruits the adaptor protein containing pH domain (APPL1) which in turn activates AMP-activated protein kinase (AMPK) [33]. These molecules also modulate phosphoinositide-3-kinase protein kinase B (PI3K/AKT) and mammalian target of rapamycin (mTOR) which function as regulatory hubs in both metabolic and immune processes. Signaling cascades that polarize T cell and macrophage responses incorporate these molecules [34, 35]. Therefore, adiponectin can regulate both the acquired and innate arms of the immune responses.

The renin–angiotensin system (RAS) has been traditionally associated with systemic blood pressure and renal electrolyte homeostasis. Mounting evidence shows that RAS plays an important role in adipose tissue inflammation [36]. Visceral adipose tissue expresses all the components of RAS. Angiotensin II is generated through the successive cleavage of angiotensinogen by renin and angiotensin-converting enzyme (ACE). Engagement of angiotensin receptor 1 (AT1r) by angiotensin II can induce several T helper-1 (Th1) cytokines leading to vascular inflammation. Furthermore, AT1r signaling can induce expression of MCP-1 and CCR2 that promote visceral adipose tissue inflammation and vascular endothelial damage [37]. It is clear now that obesity is associated with activation of RAS and decreased production of adiponectin [38]. In fact, evidence point toward RAS overactivation in obesity and the possibility that RAS to be the link between obesity and insulin resistance. Functionally, angiotensin II plays a role in energy sensing, as well as modulating fat mass expansion via its effect on adipogenesis, lipogenesis, and lipolysis. It is plausible that in a state of acute energy influx to the adipose tissue, angiotensinogen production leads to increased local angiotensin II levels, which in turn induces local vasoconstriction and lower lipolytic rates. Conversely, in fasting conditions, due to lower local angiotensin II levels, vasodilatation occurs, leading to increased rates of lipolysis. Taken together, the net paracrine effect of angiotensin II is to reduce lipolysis and promote lipogenesis, ultimately increasing lipid storage and inflammation in adipose tissue [39]. In turn, blockade of the RAS system can increase the anti-inflammatory adipokine adiponectin [40] and modify the relative balance of these two adipokines, effect that could potentially lessen visceral fat inflammation.

## ADIPOSE TISSUE IMMUNE RESPONSE

Expansion of adipose tissue is accompanied by chronic low-grade inflammation that primes target organs for the development of obesity-associated chronic inflammatory diseases. Adipose tissue-resident immune cells play a major role in the induction and regulation of obesity-induced systemic inflammation. These can be proinflammatory immune cells (e.g., neutrophils, dendritic cells, M1 macrophages, Th1 cells, B cells, and mast cells) as well as anti-inflammatory immune cells (e.g., regulatory T cells, Th2 cells, M2 macrophages, and eosinophils). Although most types of immune cells are already present in the adipose tissue, their number increases dramatically with the progression of obesity.

### Granulocytes in Adipose Tissue Inflammation

Neutrophils present fundamental mechanisms of effector cells (e.g., opsonization, agglutination, complement activation, regulation of inflammation) and participate in initiation of immune response and resolution of inflammation. Low circulating adiponectin level characteristic to obesity was shown to induce neutrophil activity and number in the peripheral blood [41]. Activated neutrophils infiltrate adipose tissue early during diet-induced obesity in mice in an attempt to limit the local inflammatory process [42]. Moreover, *in vitro* studies have shown that neutrophils physically bind adipocytes in a CD11b/ICAM-1 interaction and in a manner dependent on their activation state [42]. A recent study evidenced that diet-induced obesity in mice determined a rapid increase in adipose tissue’s neutrophil presence, lasting up to 90 days, and a parallel increased expression in the activity of neutrophil elastase [42]. Neutrophil elastase seemed to influence the following macrophage infiltration and M1 polarization, since M2 (alternatively polarized) macrophages were prevalent in obese mice lacking this enzyme [43].

Mast cells are important sensors of acute inflammation triggered by pathogenic bacteria and also play an important part in allergic type reactions [44]. More recent evidence implicates these cells in cardiometabolic diseases [45]. Ironically, when Paul Ehrlich described them in 1878, he coined them “Mastzellen” or “fattening” cells based on their granule-enriched cytoplasm. Mast cells share a common bone marrow precursor with basophil granulocyte. Both cell types respond to IgE stimulation following an allergen encounter, and they release similar mediators responsible for local and systemic anaphylactic reaction [46, 47]. As opposed to basophils, mast cells leave the bone marrow in an immature state and then fully differentiate in specific tissue sites. Thus, mast cells display tissue specificity and are more intimately related to specific homeostatic and pathologic states. Mast cells respond to microenvironment by releasing preformed contents of granules (histamine, heparin, tryptase, and chymase) or *de novo* synthesis of proinflammatory cytokines such as IL-6, IL-8, and TNF-α. Based on protease content, we distinguish either tryptase or tryptase/chymase-expressing mast cells. In terms of localization, mast cells are found in two main compartments: mucosal surfaces and perivascular connective tissue. Mast cells grow and proliferate in response to growth factors, stem cell factor (SCF), and nerve cell growth factor (NGF) as well as cytokines (IL-3, IL-4, IL-9, IL-10). Abnormal expansion of visceral adipose tissue is accompanied by influx of immune cells. Mouse models of diet-induced obesity showed accumulation of mast cells in adipose tissue [45, 48, 49]. Mast cell Kit^W-sh/W-sh^-deficient mice, lacking only mature mast cells, are resistant to diet-induced obesity and are able to maintain glucose homeostasis when fed with a high-fat diet. Analysis of their visceral adipose tissue revealed a significant reduction in proinflammatory cytokines and chemokines [48] and a decrease in macrophage number. Therefore, it appears that mast cell arrival in adipose tissue precedes the release of proinflammatory mediators that attract macrophages. Furthermore, even the pharmacological inhibition of mast cell degranulation reproduced the metabolic phenotype seen in Kit^W-sh/W-sh^-deficient mice.

Human adipose tissue appears to contain both tryptase and tryptase/chymase-expressing mast cells. Despite similar representation in both lean and obese subjects, it appears that mast cells in the latter group have an increased rate of degranulation [50]. Moreover, obese subjects that progressed to complications like diabetes were found to have a higher number of mast cells. Visceral fat mast cells from obese patients were found to produce significantly higher proinflammatory cytokines (IL-1, IL-6) and macrophage chemoattractant (MCP-1) previously shown to induce insulin resistance [51, 52]. Adipose tissue fibrosis has been linked to obesity insulin resistance and abnormal cytokine/adipokine secretion from adipose cells [45, 53]. Development of obesity in diabetic *db*/*db* mice was associated with recruitment of immature mast cells and whose maturation paralleled the metabolic abnormalities. Mast cell-derived tryptase was found to promote collagen 5 mRNA expressions in fibroblasts and was associated to adipose tissue fibrosis in *db*/*db* mice [45]. Antifibrotic compounds (tranilast, angiotensin-converting enzyme inhibitors, and silymarin) coupled with dietary interventions could prevent mast cell maturation and degranulation to reduce associated metabolic abnormalities [53].

Recently, the effects of adipose tissue eosinophils have also been documented on local macrophage activity and polarization. In the adipose tissue, alternative (M2) activation of macrophages is driven by the cytokine interleukin-4 (IL-4). Eosinophils are the major IL-4-expressing cells in white adipose tissues of mice. In their absence, the M2 macrophage number is greatly attenuated leading to impaired glucose tolerance and insulin resistance [54]. Thus, recent studies suggest that beyond monocytes and macrophages, plenty of other myeloid cells, such as dendritic cells, lymphoid cells like NK cells, NKT cells, B and T lymphocytes, and eosinophils, could play a combined role in the inflammatory process associated with obesity. Due to the presence of such an immune cell spectrum, several researchers consider adipose tissue as an ancestral lymphoid organ where physiologic and pathologic immune processes can take place simultaneously [55].

### Dendritic Cells, Monocytes, and Macrophages in Adipose Tissue Inflammation

Dendritic cells (DCs) are specialized, heterogeneous group of mononuclear cells able to acquire, process, and present antigens to naïve T cells. Based on their phenotype and functional characteristics, DCs can be found in almost all tissues and are further divided into the following: conventional/myeloid DCs (CD11^+^), plasmacytoid DCs (CD11c^−^CD303^+^), and a novel group of inflammatory DCs (inf-DCs) generated from *in situ* activation of monocytes recruited into the site of inflammation. Several studies consider obesity-induced adipose tissue hypoxia and elevated level of plasma free fatty acids (FFAs) as potential initiating events in the activation and recruitment of DCs into the enlarged adipose tissue. Bertola et al. [56] showed for the first time the accumulation of specific inflammatory dendritic cells CD11c^high^F4/80^low^ in the adipose tissue of obese mice and CD11c^+^CD1c^+^ in the adipose tissue of obese patients. The emergence and expansion of CD11c^high^F4/80^low^ DCs the in obese mice and CD11c^+^CD1c^+^ in the obese patients induced proinflammatory Th17 cell responses and macrophage accumulation and correlated with higher BMI and insulin resistance. Mice lacking DCs had a reduced number of macrophages in the adipose tissue, whereas DC replacement in DC^−/−^mice increased macrophage populations in the adipose tissue. Moreover, lean wild-type mice that received bone marrow-derived DCs had macrophage infiltration in the adipose tissue, while mice lacking DCs completely were resistant to the high-fat diet weight gain and metabolic abnormalities [57]. Importantly, Hagita et al. [58] proved that adipose tissue location can dictate the degree of associated vasculature inflammation. In an *in vivo* study, they showed that mice that had lean visceral fat transplanted around the femoral artery presented increased vascular inflammation (leukocyte and DC recruitment to the femoral artery) as compared to mice that had lean subcutaneous fat transplanted around femoral artery. Moreover, when they are used for transplantation, the visceral/subcutaneous fat from donor mice fed with a high-fat diet, the inflammatory response at the femoral artery level was substantially increased. Therefore the effect of high-fat diet on adipocytes is compartment specific [58].

New studies have shown that in response to high-fat diet, the hypertrophied adipocytes produce more CCL20, a chemoattractant whose receptor—CCR6—is highly expressed on adipose tissue dendritic cells. In addition, the adipose tissue dendritic cells express higher levels of IL-6, TGF-β, and IL-23 [59]. These are essential cytokines for Th17 cell proliferation and differentiation. Co-cultures of adipose tissue dendritic cells and naïve T cells promoted proinflammatory Th17 cell differentiation and IL-17 production. This effect was significantly increased when compared with dendritic cells derived from spleen [59]. These studies show that adipose tissue DCs are among the first to sustain the expanded adipose inflammatory milieu. Furthermore, by recruiting and activating other immune cells, including monocytes and macrophages, the adipose tissue DCs propagate the immune response associated with adipose tissue expansion [57].

Monocytes are also heterogeneous for phenotype and function, and different subsets rise in response to microenvironment cues. Two main monocyte subsets may be distinguished based on their expression of specific receptors: in humans CD14 (LPS receptor) and CD16 (FcgammaRIII) and in mice Ly6C and Gr1. Based on the relative expression of CCR2 and CX3C chemokine receptor 1 (CX3CR1), Ly6C^hi^ monocytes are Gr1^+^CCR2^+^CX3CR1^lo^ and correspond to human CD14^++^CD16^−^ (classical monocytes) whereas Ly6C^lo^ monocytes are Gr1^−^CCR2^−^CX3CR1^hi^ and correspond to human CD14^+^CD16^+^ (nonclassical monocytes). Circulating nonclassical monocytes demonstrate a patrolling behavior along blood vessel walls [60] and form “standby” deposits in noninflamed peripheral tissues such as spleen, lung, and liver [61].

However, despite the overall conservation, the comparison of the two species’ subsets highlighted some diversity such as expression of fatty acid translocase (FAT/CD36), tetraspanin CD9, triggering receptor expressed on myeloid cells 1 (Trem-1), and PPARγ. Recently, Shantsila et al. [62] demonstrated unequivocally that human monocyte group includes three major functionally and phenotypically different subsets: the classical CD14^+^CD16^−^CCR2^+^, the intermediate CD14^+^CD16^+^CCR2^+^, and the nonclassical CD14^dim^CD16^+^CCR2^−^ monocytes.

Most of the monocytes are CD14^+^CD16^−^ and can amount to up to 85 % in healthy subject [62]. The CD16^+^ monocytes increase their frequency in response to chronic inflammatory conditions, such as chronic kidney disease (CKD) [63], obesity [64, 65], and associated cardiovascular diseases [66]. High levels of the CD14^+^CD16^+^ subset of CD16^+^ were associated with cardiovascular events [67] and reduced survival at 35 months in CKD patients [68]. In the same time, CD14^dim^CD16^+^ subtype was positively correlated with the BMI [65] and atherogenic lipoproteins and inversely associated with high-density lipoprotein cholesterol.

Poitou and colleagues [69] investigated the frequency of CD16^+^ monocyte subsets and their potential role in obesity and weight loss; they showed an increase in CD14^dim^Cd16^+^ monocytes in obese and diabetic patients. Importantly, weight loss as well as surgery-induced weight loss caused a reduction of CD14^dim^CD16^+^ monocytes that correlated with reduction of subclinical atherosclerosis, as evaluated by intima–media thickness.

Obesity promotes the mobilization of monocytes from the bone marrow in part by activating the CCR2. Deficiency of CCR2 or its ligand, MCP-1, in mice results in failure of monocyte mobilization and is associated with protection from monocyte infiltration into adipose tissue and insulin resistance [70, 71]; Spite et al. [72] report that activation of the leukotriene B4 (LTB4) and its receptor BLT-1 axis is required for obesity-induced increases in peripheral blood monocytes and subsequent adipose tissue macrophage accumulation.

Adipose tissue macrophages are the main component of adipose tissue immune cells (40–60 % of all adipose tissue immune cells), and their number increases progressively after only 1 week of high-fat diet feeding [73]. Two major macrophage phenotypes have been described: classically activated or M1, which trigger a proinflammatory, type 1 immune response, and alternatively activated or M2, which promote anti-inflammatory, type 2 responses during the healing process [74, 75]. However, *in vivo*, monocytes and macrophage phenotypes more likely represent points on a spectrum with high plasticity that shapes obesity-induced inflammation.

Progressive adipose tissue expansion is accompanied by macrophage accumulation and decreased expression of key genes of adipocyte differentiation (PPARγ and C/EBPα). This reduces the number of new, small adipocyte recruitment and leads to mature adipocyte hypertrophy [76]. Nevertheless, there are different rates of macrophage accumulation based on the anatomical location of the adipose tissue (i.e., visceral vs subcutaneous fat). Human subcutaneous adipose tissue macrophages retrieved by liposuction from healthy, overweight women are composed mainly of cells expressing CD206, a marker of activated macrophages. However, it seems that only the CD206^+^/CD16^+^ cells accumulate in the adipose tissue directly proportional with adiposity. Although a rapid local differentiation of inflammatory monocytes into macrophages cannot be excluded, enhanced local proliferation might be involved in the accumulation of CD206^+^/CD16^+^ cells.

Significant differences in MCP-1 production and in the amount of infiltrated macrophages were found in the subcutaneous, epididymal, renal, and mesenteric fat samples from obese and control mice. The MCP-1 protein levels were significantly higher in the obese mice than those in the nonobese controls, with the highest MCP-1 level detected in the mesenteric adipose tissue sample from obese mice. Moreover, the differences in MCP-1 level among anatomically different adipose tissues correlated with the number of macrophages infiltrated into that fat pad. These results indicate that the mesenteric adipose tissue is a major depot for MCP-1, which can modulate macrophage trafficking and activation during obesity-related inflammatory diseases [77]. On the other hand, an experimental study in mice evidenced new players such as microRNAs (miR-233) that suppress the classic proinflammatory (M1) pathways and enhance the alternative anti-inflammatory (M2) responses in the adipose tissue [78].

### Natural Killer Cells and Natural Killer T Cells in Adipose Tissue Inflammation

Obesity is accompanied by a low-grade, systemic inflammatory process that involves both innate and adaptive immunity.

High-fat diet feeding stimulates the secretion of interferon gamma (IFN-γ) in the adipose tissue of wild-type mice. Several studies showed that IFN-γ initiates early accumulation of T and B lymphocytes in the adipose tissue and activates local macrophage recruitment and their classical M1 differentiation [79]. In humans, O’Rourke et al. [80] showed that visceral adipose tissue from obese individuals presented elevated IFN-γ transcript levels and a high frequency of macrophages, T cells, and natural killer (NK) cells relative to subcutaneous adipose tissue. On the other hand, obese but IFN-γ-deficient mice had significantly less adipose tissue expression of inflammatory genes such as TNF-α and MCP-1 and better glucose tolerance than the obese, control mice consuming the same diet [81]. Moreover, the absence of T and B lymphocytes in the RAG2^−/−^mice fed with a high-fat diet had no effect on the increased macrophage accumulation in the expanded adipose tissue or insulin resistance [79]. In conclusion, in the absence of T and B cells, the NK cells are also able to produce IFN-γ and TNF-α, which are relevant to macrophage recruitment in the adipose tissue during obesity.

The early role of natural killer T (NKT) cells and their regulation in adipose tissue immune response are not yet thoroughly deciphered. Currently, it is known that NKT cells can bridge innate and adaptive immune responses [82]. There are three types of NKT cells: invariant NKT (iNKT), noninvariant NKT, and NKT-like cells. Invariant NKT and noninvariant NKT cells are CD1d dependent [83]. The MHC class I-like CD1d glycoprotein is a member of the CD1 family of antigen-presenting molecules and is responsible for the selection of NKT cells. Importantly, NKT cell population and CD1d expression was found to be highly expressed in adipocytes from obese mice and humans compared to those from lean mice and lean human subjects [84]. In addition, the CD1d-expressing adipocytes are able to stimulate NKT cell activity through mere physical interaction. In animal studies, CD1d^(−/−)^ mice fed with a high-fat diet gained little weight, had less liver inflammation, and presented smaller adipocytes in comparison with wild-type control mice on the same diet [85]. The NKT cell-deficient Jα18^−/−^mouse model fed with a high-fat diet became more obese and displayed increased adipose tissue inflammation in the early stage of obesity. These results underline the role of NKT in the early adipose tissue inflammation and obesity-related insulin resistance [73, 84].

### T cells in Adipose Tissue Inflammation 

Adaptive immunity seems to assign a causative role to B and T lymphocytes in activating innate immunity [86], while regulatory T (Treg) cells have a suppressive function, rescuing obese mice from chronic adipose tissue inflammation [87].

Duffaut et al. [79, 88] observed that fat expresses a predominant macrophage population with CD3^+^-activated T cells (including CD4^+^ T helper and CD8^+^ T cytotoxic cells), a minor number of CD56 NK cells, and few CD19^+^ B lymphocytes. The CD3^+^CD56^+^ NKT cells and CD25^+^ Treg cells were found in a very low number in steady state. Interestingly, most CD3^+^-activated T cells were organized in clusters surrounding adipocytes, and their number increased proportionally with the adipose tissue size and BMI. However, this distribution was influenced by the degree of obesity and by adipose tissue location. Visceral adipose tissue from obese patients showed an increased number of macrophages and lymphocytes, especially CD8^+^ effector T cells, compared to subcutaneous fat. Moreover, proinflammatory chemokines followed a similar pattern and increased proportionally to the amount of visceral adipose tissue [79, 88]. Both CD4^+^ and CD8^+^ T cells have been found in adipose tissue, and their number increases with obesity [81] in both humans and mice [89]. Depletion of CD8^+^ cells in obese mice decreased the number of macrophages in adipose tissue and lowered TNF-α and IL-6 levels, while T cell receptor (TCR)^−/−^mice were clearly protected against obesity-induced hyperglycemia and insulin resistance [90]. Accordingly, adoptive transfer of CD8^+^ cells induced M1 macrophage accumulation, impaired glucose tolerance, and insulin sensitivity in obese mice [86].

Fabbrini et al. showed that adipose tissue from insulin-resistant obese patients had 3- to 10-fold more CD4^+^ T cells that produced IL-22 and IL-17 in comparison with adipose tissue from insulin-sensitive obese and lean subjects. IL-17 and IL-22 inhibited uptake of glucose through receptors for IL-22 and IL-17 expressed in the human liver and skeletal muscle [91].

Treg cells are thought to maintain tolerance/anti-inflammatory microenvironment through IL-10 production. Tregs are abundant in visceral adipose tissue of lean mice, but their number is significantly reduced in insulin-resistant mice models of obesity [87]; similarly, a reduced number of Foxp3^+^ Treg cells was found in visceral adipose tissue from obese humans [89]. The signal transducer and activator of transcription 3 (STAT3) plays an important role in the Th1/Treg balance within the adipose tissue. STAT3 activity is increased in visceral adipose tissue of obese mice and is also associated with increased IL-6 production, an inhibitor of Treg function. Ablation of STAT3 suppresses adipose tissue inflammation, increases the ratio of Treg/Th1 cells, and promoted M2 macrophage accumulation [92]. It has recently been found that Tregs express the insulin receptor and that stimulation with high levels of insulin induces a decrease in their IL-10 production through activation of AKT signaling, thus contributing to obesity-associated inflammation. Moreover, the hyperinsulinemic mice fed with a high-fat diet showed a significant decrease in visceral adipose tissue Tregs IL-10 production and an increase in IFN-γ production [93]. Lifestyle, nutritional, and pharmacological interventions aimed at restoring insulin sensitivity may also restore the Treg function in obese patients.

Interestingly, Poutahidis T. et al [94] found an association between western diet-associated obesity, type of gut microflora, and CD4^+^ Th17 prevalent T cell phenotype. Display of proinflammatory immune cell profile was prevented by microbial targeting that induced Foxp3^+^ regulatory T cells and IL-10. Taken together, these findings support interventions aimed to enhance the anti-inflammatory properties of Tregs in humans and reduce the development of obesity-associated inflammation.

A novel subset of T helper cells, Th22 has been linked to chronic inflammatory conditions including obesity and diabetes. The proportion of circulating Th22 cells is increased in overweight/obese patients. Consistent with this observation, serum IL-22 level was significantly increased in obese patients when compared with lean subjects. The development of diabetes within the obese patient population led to further increase in circulating Th22 cells and IL-22 [95] emphasizing the potential association between Th22 and the pathogenesis of obesity and type 2 diabetes.

## NOVEL TRIGGERS OF ADIPOSE TISSUE IMMUNE RESPONSE

### Aryl Hydrocarbon Receptor Agonists

The rapid increase in the number of people with obesity and obesity-induced chronic inflammatory diseases is now attributed to intricate cross talk between genetic makeup and so termed environmental “obesogens” [96]. Among these, more than 20 chemicals have been shown to cause long-term weight gain based on exposures during critical periods of development. Smoking and nicotine, persistent organophosphate pesticides, flame retardants, plasticizers and plastics, and fungicides, for example, have all been linked to obesity in animals. These highly lipophilic toxicants have very long half-lives that allow them to accumulate in the food chain. Western style diet, based on high consumption of animal fat, increases human exposure to these ubiquitous toxicants. The dioxin and dioxin-like pollutants are among the most dangerous. Due to their long half-life and lipophilicity, they accumulate in adipocytes and participate in the pathophysiology of obesity and obesity-associated chronic inflammatory diseases [97, 98] through activation of the aryl hydrocarbon receptor (AhR) [99, 100]. AhR is a ligand-activated transcription factor with important roles not only in the xenobiotic metabolism but in developmental and normal physiology as well. This particular receptor is ubiquitously present in adipocytes and, most importantly, in all the cells that participate in the immune system responses [101, 102]. Moreover, the preadipocytes that differentiate into mature adipocytes in the presence of even low levels of these toxic AhR ligands produce significantly more inflammatory cytokines such as TNF-α, IL-6, and chemokine MCP-1 [103]. Long-term exposure of mice to dioxin-like AhR agonists led to increased visceral adipose tissue mass, ectopic fat deposition in the liver (hepatic steatosis) and peritoneal cavity, and abnormal serum lipid profile similar with the metabolic syndrome [98]. Importantly, under the same treatment, AhR KO mice appear resistant to obesity and its metabolic consequences. Consistent with these observations, ApoE^−/−^mice that received dioxin-like PCBs (AhR agonists) developed atherosclerosis, as early event in the pathogenesis of abdominal aortic aneurysms (AAAs) [98].

Inflammation plays an important role in the development of atherosclerotic lesions and aortic aneurysms. Proinflammatory cytokines can be released systemically or produced locally within the endothelium, aortic wall, or, more importantly, in the inflamed periaortic fat. In a mouse model of angiotensin-induced AAA, obese ApoE^−/−^mice had higher expression of MCP-1 and macrophage infiltration in the perianeurysmal fat tissue when compared with lean ones [104]. Collectively, *in vivo* studies showed that dioxin-like compounds increase expression of proinflammatory cytokine TNF-α, chemokine KC (CXCL1), and MCP-1 within adipocytes in the inflamed perianeurysmal adipose tissue.

Obesity is associated with migration of bone marrow-derived macrophages into the visceral adipose tissue where they acquire an M1 (classical activation) phenotype and secrete proinflammatory cytokines such as IL-1, IL-6, and TNF-α [105]. Mice exposed to dioxin-like toxicants presented a significant increase in CD68^+^ cells in the aneurismal sac and surrounding fat tissue consistent with a macrophage infiltrate [104]. On the other hand, AhR pathway is involved in the activation of the RAS. Infusion of angiotensin II induces aneurysm formation in mice prone to atherosclerosis (i.e., ApoE^−/−^, LDL^−/−^). Adding dioxin-like toxicants (PCBs) to the angiotensin II infusion increased the incidence and severity of the aneurysms and had an additive effect, proportional with the visceral fat mass. This effect is maintained by downregulating the production of the anti-inflammatory adiponectin. This adipokine secretion decreases with the increased body mass, contributing to the chronicity of the adipose tissue inflammatory process.

### Uremic Toxins

CKD is prevalent in the general population. CKD is associated with adverse outcomes such as cardiovascular mortality and morbidity. Diabetes and obesity are some of the main factors associated with higher risk of CKD. Recently, it has been shown that the circulating levels of MCP-1 are higher even in “healthy” obese individuals with an apparently unaffected renal function than those in normal weight control subjects. This situation is especially critical due to the association between plasma level of MCP-1 and of cystatin C, as well as a correlation between urinary MCP-1 and creatine-to-cystatin ratio, indicating the existence of a subtle, early kidney injury in otherwise healthy obese individuals [106]. Mounting evidence supports a strong association between increased body weight and CKD and its progression to end-stage renal failure. Excessive caloric and protein intake has long been considered to facilitate kidney failure in individuals with CKD. Indoxyl sulfate (IS) and *p*-cresyl sulfate (PCS) are two novel toxins solely produced by degradation of dietary tyrosine, phenylalanine, and tryptophan by the gut microbiota and further metabolized in the liver that are present in high concentrations in CKD patients. IS and PCS are gut-generated uremic toxins that circulate predominantly bound to albumin. They associate with a systemic inflammatory milieu (high serum IL-6, TNF-α, IFN-γ). They are difficult to remove by dialysis, and their free fraction accumulates in the serum proportionally with the CKD stage. Recent experimental studies have shown that these protein-bound toxins are involved not only in the progression of CKD but also in the aggravation of cardiovascular disease [107]. While PCS had proinflammatory effects on nonstimulated leukocytes *in vitro* and contributed to vascular damages, IS inhibited endothelial cell repair through induction of reactive oxygen species (ROS) and activation of the NF-kB pathway [108]. Moreover, IS can upregulate expression of MCP-1 and tissue factor in endothelial cells and macrophages through activation of the AhR, a xenobiotic sensor that mediates adaptive and toxic responses in cells [109]. Increasing premorbidity due to obesity and insulin resistance coupled with gut western dietary intake substrate will reverberate in an increase gut-derived toxins and endobiotics. On the other hand, chronic exposure to dioxin-like pollutants (AhR ligands) through a high-fat diet and their accumulation in the adipose tissue will also play an important role in the overproduction of gut-derived toxins, contributing to the increased cardiovascular risk in CKD patients [110] (Fig. 1).

## ADIPOSE TISSUE INFLAMMATION: CONSEQUENCES FOR CHRONIC INFLAMMATORY CONDITIONS

### Abdominal Aortic Aneurysms

AAA is defined as an aortic dilation of 3.0 cm or more in either anteroposterior or transverse planes [111]. In many cases, AAA rupture may be the first clinical manifestation of the abdominal aorta pathology. Ruptured AAA carries a mortality risk of 70 % among patients that reach the hospital. Although it is very well known that smoking, advanced age, and male gender are associated with a higher prevalence of AAA, the etiology remains uncertain, and recent evidence points toward an increased role of adipose tissue immune response [112]. Although the vascular pathology in AAA patients is believed to develop through mechanisms distinct from atherosclerosis, it is many times associated with obesity and obesity-related conditions.

**Fig. 2:**
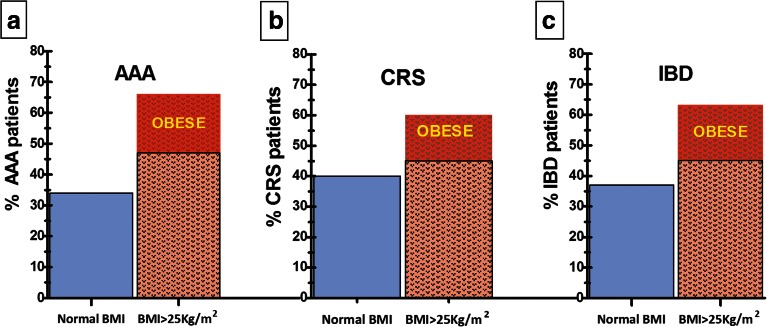
High prevalence of overweight and obesity in patient populations with chronic inflammatory conditions. Obesity has become a worldwide epidemic. Over two thirds of patients diagnosed with chronic conditions such as inflammatory bowel diseases, cardiorenal syndrome, or abdominal aortic aneurysm in our clinics during the last 2 years were overweight or obese.

We analyzed a cohort of 197 consecutive AAA patients that requested elective surgery for AAA repair between September 2007 and October 2011 in the Vascular and Endovascular Surgery Unit at the S. Martino University Hospital, University of Genoa, Italy. Two thirds of these AAA patients were overweight/obese (47 % were overweight (BMI 25–29.9 kg/m^2^) and 19 % were obese (BMI ≥30 kg/m^2^)) (Fig. 2a). While only 39 % of our patients were current smokers, the majority (93 %) reported smoking over 100 cigarettes in their lifetime. In these aspects, our data correlate with other studies that show positive association with increasing years of smoking and cigarettes smoked, as well as a positive association of AAA with excess weight. Importantly, a preferential abdominal localization of visceral fat, rather than general obesity, may be more relevant to the etiology of AAA [113] as waist circumference (WC) and waist-to-hip ratio (WHR) were found in several studies to have a significant positive association with AAA [114]. Locally, the perivascular and perianeurysmal adipose tissues have been shown to affect inflammation, formation, and severity of experimental AAA in animals [104, 115–117]. In a study that included 48,850 men and 39,227 women, Stackelberg et al. concluded that individuals with an increased WC (>100 cm for men and >88 cm for women) had a 30 % higher risk of AAA compared with those with a normal WC. Moreover, the intra-abdominal fat assessed by WC correlated with periaortic adipose tissue mass [118]. Total adiposity expressed as BMI had no significant correlation with the AAA incidence and progression. Interestingly, the AAA diameter decreased with weight loss in mice, limiting the AAA progression [104].

Mechanistically, the enlarged periaortic adipose tissue produces proinflammatory cytokines such as IL-6, IL-8, and MCP-1 that aggravate vascular inflammation, while the secretion of anti-inflammatory adiponectin is markedly reduced. High-fat feeding will further reduce the secretion of adiponectin in human perivascular adipocyte, while upregulating several proinflammatory gene expression [119, 120]. This is an important stage of the inflammatory process because adiponectin is an endogenous modulator of vascular remodeling proven to abolish proliferation and migration of human vascular smooth muscle cells (hVSMC) [121] by directly binding to platelet-derived growth factor-BB-binding protein (PDGF-BB) [122]. Adiponectin knockout mice exhibited severe neointimal thickening and increased proliferation of VSMC in mechanically injured arteries and exhibited profound neointimal hyperplasia [123]. In other animal studies, the increased expression of inflammatory cytokines was found to trigger the infiltration of macrophages in the perivascular adipose tissue followed by increase formation and growth of AAA. Ohashi et al. showed that adiponectin modulates macrophage polarization toward the alternatively activated M2 cells. Macrophages collected from adiponectin knockout mice displayed increased M1 markers (TNF-α, IL-6, and MCP-1) and decreased M2 markers (IL-10, arginase-1, and macrophage galactose *N*-acetyl-galactosamine-specific lectin-1). Macrophages from both wild-type and adiponectin knockout mice switched their polarization toward M2 activation after overexpressing the adiponectin. Monocyte-derived macrophages isolated from human adipose tissue behaved the same after treatment with recombinant adiponectin promoting the anti-inflammatory phenotype [124].

Moreover, Kent et al. showed that over 55 % of AAA patients followed by his team had a diet poor in fruit, vegetables, and nuts but rich in fat and processed meats (western diet) [112]. This is an important aspect since organic pollutants are persistent, lipophilic, and bioaccumulate in the food chain. High-fat foods and highly processed fatty meats are the main ways of human chronic exposure to these toxicants followed by accumulation in human adipose tissue. An increased concentration of persistent organic pollutants in the visceral fat as well as in perianeurysmal fat has been shown to contribute to the AAA incidence and growth in animal studies [98]. In humans, bariatric surgery is used to limit the amount of ingested food. Patients that underwent bariatric surgery show restoration of perivascular adipose tissue vasodilatory capacity, reduction of perivascular inflammation, and oxidative stress with improved adiponectin and nitric oxide bioavailability in the perivascular adipose tissue [125].

### Cardiorenal Syndrome

Cardiorenal syndrome (CRS) represents a complex cluster of conditions and clinical presentations of combined heart and kidney disorders. Direct and indirect effects of each organ that is dysfunctional can initiate and perpetuate the combined disorder of the two organs through a complex combination of neurohormonal feedback mechanisms [126]. An effective classification of the CRS was proposed by the Italian nephrologist Claudio Ronco in 2008. CKD patients with increased plasma levels of high sensitivity C-reactive protein (hs-CRP), IS, PCS, or serum amyloid A protein have a higher rate of cardiovascular events. Overweight patients with heart failure (HF) are ideal candidates to develop CRS and to suffer from local and systemic inflammation. Excess body weight has been associated with elevated systemic inflammatory markers, such as hs-CRP or IL-6 that contribute to tubular lipid accumulation and pervasive inflammation characteristic to CKD [127, 128]. In other studies, obesity itself was an independent risk factor for the development of CKD [129]. The interaction between obesity and other renal disease-promoting factors has been partly elucidated through several observational and mechanistical studies. One of the potential mechanisms by which obesity promotes CKD is through hyperfiltration-related maladaptive mechanisms. In one study [130], investigators phenotyped 1,572 young men for various metabolic risk factors. Renal function was ascertained by calculating creatinine clearance (Cockcroft–Gault equation). The early renal functional abnormalities were associated with adiposity (elevated leptin levels and high BMI) and blood pressure. Increased adiposity, and in particular abdominal visceral fat, led to an enhanced production of inflammatory adipokines and glomerular hyperfiltration early in the disease. In addition, the pro-oxidant and proinflammatory state that accompanies insulin resistance in overweight and obese patients with CRS may trigger activation of the RAS and further increase the proinflammatory molecules produced by the liver and by the adipose tissue [131, 132]. Studies in obese angiotensin receptor 1a knockout (AT1a-KO) mice fed with a high-fat diet showed increased visceral fat and kidney macrophage infiltration with a prevalent proinflammatory M1 phenotype. The obese AT1a-KO presented increased mesangial expansion, tubular vacuolization, and downregulated M2 macrophage markers compared with lean mice. Treatment with AT1 receptor blocker abolished renal macrophage infiltration and switched the macrophage polarization toward the M2 anti-inflammatory and reparatory macrophage phenotype [133].

Starting from September 2010 to October 2011, we collected data from 104 male patients with stable chronic HF NYHA class I–II–III with ejection fraction ≤45 % at the time of their first visit in the outpatient congestive heart clinic at S. Martino University Hospital, Genoa, Italy. Sixty percent (60 %) of our male patient cohort with CRS were overweight or obese (45 and 15 % respectively, Fig. 2b). Out of 104 patients, 93 were former smokers and 11 patients were current smokers. As far as renal damage, 35 % had mild (CKD II), 45 % had moderate (CKD III), and 7 % had severe (CKD IV) decreases in the glomerular filtration rate (GFR). Higher BMI correlated to decreased eGFR (Pearson correlation coefficient *r* = 0.267 and *p* = 0.025). In addition, higher BMI correlated with higher values of circulating uric acid (*r* = 0.277, *p* = 0.033). Our data correlate with other studies showing that tissue injury in both kidney and HF in the context of obesity has immune-mediated inflammatory consequences that can accelerate remote organ dysfunction.

### Inflammatory Bowel Diseases 

Crohn’s disease pathogenesis as an inflammatory bowel disease (IBD) is considered to be an inappropriate immune response to the luminal bacteria. Although a primary epithelial defect is believed to set in motion the innate and acquired arms of the immune system, a signature feature of Crohn’s disease is the development of mesenteric fat inflammation. Macroscopically, the fat tissue wraps around the diseased bowel segments, enveloping them in so called “creeping fat.” Given the transmural nature of the inflammation in IBD, the mesenteric fat inflammation has generally been considered a secondary event. We collected anthropometric data from 634 consecutive IBD patients seen in the Inflammatory Bowel Diseases Center at The Ohio State University, USA. Sixty-three percent (63 %) of them were overweight and obese (45 and 18 %, respectively) while only 37 % had a normal body weight (Fig. 2c). Our data, as well as other recent epidemiological studies, show an increase incidence of Crohn’s disease patients that are overweight and obese [134]. Due to the intimal involvement of the mesenteric fat to the intestinal inflammation present in IBD, it is reasonable to assume that adiposity plays an important role in initiating and perpetuating intestinal inflammation. Emerging data from multiple medical fields clearly demonstrate that adipocytes and resident adipose tissue macrophages function as bacterial sensors and participate firsthand in the inflammatory process. Moreover, both macrophages and adipocytes share regulatory pathways relevant to metabolism and innate immunity [135]. The small intestine is able to adapt its lipid absorption capacity to the fat content of the diet connecting the intestinal lipid metabolism with the susceptibility to obesity [136]. Excess body weight is associated with systemic microinflammation, and adipose tissue is a known source of proinflammatory cytokines (angiotensin, TNF-α, IL-6) [137]. The expanded, inflamed “creeping fat” can thus become a source of inflammatory mediators at the expense of the anti-inflammatory adipokine and adiponectin. Signaling through adiponectin receptors regulates overlapping pathways responsible for energy balance, insulin sensitivity, and macrophage polarization. Overexpression of adiponectin or treatment with an adiponectin agonist can protect mice from experimental colitis [138, 139] linking adiponectin levels with inflammation. Moreover, overactivation of the environmental sensor—AhR—was associated with worse colitis and a low ratio of adiponectin to angiotensin. The opposite was noted in AhR heterozygote mice [140] that have approximately half the AhR tissue abundance. Targeted weight loss or dietary interventions could increase HMW adiponectin in IBD patients and decrease inflammation. On the other hand, smoking cessation and diets low in fat and processed meats may decrease exposure to dioxin-like toxins with beneficial effects on mucosal and mesenteric fat inflammation.

Visceral adiposity may play an extended role in the initiation and perpetuation of inflammation in Crohn’s disease patients with cardiovascular comorbidities [134]. Looking in detail to the link between IBD and coronary artery disease, Gandhi recently reported that the presence of elevated markers of systemic inflammation predicted coronary events in IBD population [141]. Thus, due to their established pathogenic role for promoting vascular damage [142], circulating levels of markers of inflammation such as hs-CRP are likely to predict coronary artery disease in IBD patients.

## POTENTIAL STRATEGIES TO MODULATE ADIPOSE TISSUE INFLAMMATION

Traditionally, treatment for obesity can include diet, physical exercise, and pharmaceutical or surgical interventions [32]. Nevertheless, novel strategies should focus on reducing the adipose tissue inflammatory status before dieting and exercising as this approach might accelerate weight loss and increase patient compliance. Already available PPARγ agonists promote the development of small size, insulin-sensitive adipocytes although there is an initial increase in the volume of adipose tissue. Furthermore, PPARγ agonists promote the M2 macrophage polarization and stimulate the smaller, insulin-sensitive adipocytes to secrete adiponectin, thus reversing the inflammatory milieu [143]. Decreasing adipocyte size and reducing periorgan fat deposition will further help normalize adiponectin secretion [31]. Higher circulating levels of adiponectin have a significant impact on adipose tissue macrophage polarization, favoring the M2 anti-inflammatory and reparative phenotype [138].

Dual compounds like ARB/PPAR ligands (i.e., telmisartan and irbesartan) can selectively block angiotensin II type 1 receptor (AT1) [144] while selectively modulating PPAR. Both actions suppress inflammatory molecules, oxidative stress, decrease visceral fat accumulation, and augment adiponectin and leptin activities [40] In addition, this treatment will reduce fibrosis and preserve endothelial function while improving vascular and cardiac functions [145].

High-fat diet, smoking, and pollution can be blamed for adipose tissue becoming a repository of lipophilic toxic compounds [123]. It is therefore conceivable that surgically reducing the fat mass can significantly decrease the amount of toxic and lipophilic body burden and correct the immune and metabolic imbalances. In addition, dietary intervention with AhR ligands from cruciferous vegetables may block the effect of these toxins through competitive binding of this receptor.

Serum concentration of uremic toxins depends on the dietary intake, GFR, and tubular secretion. Factors promoting generation and absorption include an increased ratio of dietary protein to carbohydrates, an insufficient intake of fibers, and/or reduced intestinal protein assimilation as well as prolonged colonic transit time. Studies with prebiotic and/or probiotic therapies targeting intestinal production of IS and PCS modulated toxins production and absorption by decreasing the intestinal bacterial growth and metabolism in patients with CKD. Ingestion of a proportionally higher alkaline diet (e.g., a more vegetarian diet), low animal protein ingestion and, hence, purine intake could proportionally lower serum uric acid levels.

On the other hand, binding therapies can neutralize toxic precursors and block their intestinal absorption [146, 147]. For example, AST-120, an orally ingested activated charcoal adsorbent of uremic toxins, has been used with success in halting CKD progression. In human studies, AST-120 treatment preserved renal function in patients with early stage of CKD and type 2 diabetes and delayed progression to end-stage renal disease. Overall, early treatment of CKD patients with AST-120 resulted in a significant reduction of CV events [148, 149]. Another strategy that might be employed to reduce uremia could be modulation of the AhR-signaling pathway since the uremic toxin IS is also a potent activator of AhR [150].

Reports from the Framingham Heart study investigator showed that vitamin D deficiency is strongly associated with visceral adiposity [151]. Supplementation with oral calcium and vitamin D (orange juice) favors weight loss and a beneficial reduction in the visceral adipose tissue in overweight and obese adults [152]. This antilipolytic effect might be due to the increased intracellular calcium, decreased intracellular cAMP level, downregulated hormone-sensitive lipase (HSL), and adipose triglyceride lipase (ATGL) protein expression in adipocytes. In addition, several studies show that dietary intake of calcium and vitamin D is inversely associated with visceral adipocyte size and that higher consumption of foods that are enriched in calcium and vitamin D might help reduce the visceral adipose tissue mass and the associated metabolic disturbances.

## CONCLUDING REMARKS

There are new and viable dietary and pharmaceutical interventions that can be deployed to reduce the cardiovascular risk when amplified by adipose tissue inflammation. Fortunately, current methods that gauge and quantify adipose tissue are well established and accessible to every cardiovascular patient [153]. This review brings in the limelight several anti-inflammatory agents and interventions that are currently in clinical trials or yet to be moved from the researcher bench to the patient bed. New approaches, such as using adiponectin agonists, PPARγ agonists, dual ARBs/PPARs, and dietary modulators of the AhR in addition to a healthy diet and exercise, could rapidly reduce the adipose tissue inflammation. More studies are imperatively necessary to clearly define the patient populations who will benefit from these new therapies.
